# p58^IPK^ Is an Endogenous Neuroprotectant for Retinal Ganglion Cells

**DOI:** 10.3389/fnagi.2018.00267

**Published:** 2018-09-07

**Authors:** Todd McLaughlin, Narayan Dhimal, Junhua Li, Joshua Jianxin Wang, Sarah Xin Zhang

**Affiliations:** ^1^Departments of Ophthalmology and Biochemistry, Ross Eye Institute, University at Buffalo, State University of New York, Buffalo, NY, United States; ^2^SUNY Eye Institute, State University of New York, Buffalo, NY, United States

**Keywords:** p58^IPK^, retinal ganglion cells, retinal ischemia/reperfusion, ocular hypertension, X-box binding protein 1

## Abstract

p58^IPK^ is an endoplasmic reticulum (ER)-resident chaperone playing a critical role in facilitating protein folding and protein homeostasis. Previously, we have demonstrated that p58^IPK^ is expressed broadly in retinal neurons including retinal ganglion cells (RGCs) and loss of p58^IPK^ results in age-related RGC degeneration. In the present study, we investigate the role of p58^IPK^ in neuroprotection by *in vitro* and *in vivo* studies using primary RGC culture and two well-established disease-relevant RGC injury models: retinal ischemia/reperfusion (I/R) and microbead-induced ocular hypertension. Our results demonstrate that in both* in vivo* models, p58^IPK −/−^ mice exhibit significantly increased RGC loss compared to wild type (WT) mice. *In vitro*, p58^IPK^-deficient RGCs show reduced viability and are more susceptible to cell death induced by the ER stress inducer tunicamycin (TM). Overexpression of p58^IPK^ by adeno-associated virus (AAV) significantly diminishes TM-induced cell death in both WT and p58^IPK −/−^ RGCs. Interestingly, we find that loss of p58^IPK^ leads to reduced mRNA expression, but not the protein level, of mesencephalic astrocyte-derived neurotrophic factor (MANF), a neurotrophic factor that resides in the ER. Treatment with recombinant MANF protein protects R28 retinal neural cells and mouse retinal explants from TM-induced cell death. Taken together, our study suggests that p58^IPK^ functions as an endogenous neuroprotectant for RGCs. The mechanisms underlying p58^IPK^’s neuroprotective action and the potential interactions between p58^IPK^ and MANF warrant future investigation.

## Introduction

Accumulating evidence suggests that disturbed protein homeostasis in the endoplasmic reticulum (ER), or ER stress, is a significant contributing factor to neurodegeneration in the central nervous system including the retina (Zhang et al., 2014). Prolonged ER stress activates pro-apoptotic genes such as C/EBP homologous protein (CHOP) resulting in apoptosis, while reducing ER stress alleviates cell death of retinal neurons, such as retinal ganglion cells (RGCs), induced by a variety of disease-provoking insults (Hu et al., 2012; Jing et al., 2012; Chen et al., 2014; Huang et al., 2015). In addition, ER stress reduces the production of neurotrophic factors but increases the secretion of pro-inflammatory cytokines, which further exacerbate neuronal death (Zhong et al., 2012; Kim et al., 2014; Cai et al., 2016). Thus, restoring ER protein homeostasis may provide a new strategy to prevent and treat retinal neurodegeneration.

The ER chaperone p58^IPK^ is an important component of the ER protein folding system and is upregulated by the highly conserved unfolded protein response (UPR) genes XBP1 and ATF6 (Lee et al., 2003; van Huizen et al., 2003). Activation of p58^IPK^ facilitates protein folding, reduces ER stress, and promotes cell survival (Rutkowski et al., 2007). In pancreatic β cells, deletion of p58^IPK^ results in CHOP-mediated apoptosis and reduced insulin production (Huber et al., 2013). In a previous study, we demonstrated that p58^IPK^ is present throughout the retina and mice lacking p58^IPK^ display increased susceptibility to retinal injury caused by glutamate toxicity and age-related cell death (Boriushkin et al., 2015). In R28 retinal neural cells, p58^IPK^ overexpression suppresses ER stress and improves cell survival under conditions of oxidative stress (Boriushkin et al., 2015). Furthermore, p58^IPK^ acts as a potent inhibitor of NLRP3 inflammasome and reduces IL-1β secretion in bone marrow macrophages (Boriushkin et al., 2016). These findings suggest that p58^IPK^ may function as a neuroprotectant and play an important role in maintaining the viability of retinal neurons such as RGCs in pathogenic conditions related to ischemic retinopathy and glaucoma.

In the present study, we tested this hypothesis in two commonly used RGC injury models induced by retinal ischemia/reperfusion (I/R) or ocular hypertension and explored the potential mechanism of p58^IPK^ neuroprotection and its interaction with mesencephalic astrocyte-derived neurotrophic factor (MANF), an ER stress responsive neurotrophic factor.

## Materials and Methods

### Animals

The generation and maintenance of p58^IPK^ knockout mice were previously described elsewhere (Ladiges et al., 2005; Boriushkin et al., 2015). All experimental procedures were performed in compliance with protocols approved by the Institutional Animal Care and Use Committees at the State University of New York at Buffalo.

### Isolation and Culture of Mouse RGCs

RGCs were isolated from retinas of postnatal day 6 neonatal mice using the Miltenyi Biotec magnetic cell sorting (MACS) system following a published protocol (Huang et al., 2003; Jiao et al., 2005). Immediately after isolation, cells were incubated with adeno-associated virus (AAV)-GFP or AAV-p58^IPK^ (Vector Biolabs, Malvern, PA, USA) at 10^∧^12 GC/ml following standard procedures approved by the Institutional Biosafety Committee at the University at Buffalo. After 24 h, transduced cells were treated with 1 μg/ml tunicamycin (TM) or a vehicle control (0.05% DMSO) for an additional 16 h. Cell viability was examined using the live/dead cytotoxicity assay (Molecular Probes) following the manufacturer’s protocol. Images were analyzed for live cells (green) and dead cells (red), blind to treatment or genotype (see Supplementary Material).

### Retinal Ischemia/Reperfusion (I/R) Mouse Model

The anterior chamber of anesthetized mice was canulated with a 30g needle attached to a reservoir of sterile PBS. Retinal ischemia was induced by elevating the reservoir to generate a hydrostatic pressure of 90 mmHg for 60 min. After 7 days, mice were sacrificed and retinal whole-mounts were prepared for Tuj1 immunostaining to visualize RGCs (see Supplementary Material).

### Microbead-Induced Ocular Hypertension Mouse Model

Microbead injection was performed as described previously (Chen et al., 2011). Briefly, the anterior chamber of anesthetized mice was canulated and 2 μl PBS containing 10 μm diameter polystyrene, fluorescent microspheres at 1.5 × 10^7^ beads/ml (FluoSpheres, Invitrogen) was injected. Intraocular pressure (IOP) was recorded by bounce tonometer (Icare). Two weeks after microbead injection mice were sacrificed and retina whole-mounts were prepared for Tuj1 immunostaining (see Supplementary Material).

### R28 Cell Treatment and Analysis

R28 cells were cultured as described previously (Boriushkin et al., 2015). At approximately 60%–70% confluence, cells were pretreated with 50 ng/ml recombinant human MANF (hMANF; PeproTech) or vehicle for 16 h and then treated with 5 μg/ml TM or vehicle for 24 h. Cells were labeled using the live/dead cytotoxicity assay, photographed, and analyzed (see Supplementary Material).

### Immunohistochemistry and Morphological Analysis

Mouse eye cryosections were prepared for immunohistochemistry as described previously (McLaughlin et al., 2018). The primary antibodies used were: Ribeye (Synaptic Systems 192003, 1:800); PKCα (Santa Cruz sc-8393, 1:400); Pax6 (DSHB Pax6-s, 1:25); and calretinin (Millipore Mab1658, 1:800). The secondary antibodies used were: Texas-red conjugated goat-anti-rabbit (ThermoFisher T6391, 1:800); Alexa594 conjugated goat-anti-mouse (ThermoFisher A11005, 1:800). Photomontages were assembled with Adobe Photoshop.

### Quantitative RT-PCR

Total RNA was isolated with Trizol (Invitrogen) per manufacturer instructions. cDNA was made from 500 ng of total RNA using Bio-Rad cDNA kit followed by quantitative RT-PCR (qPCR) with Bio-Rad iQ Sybr Green Supermix. The following primers were used: MANF f1: 5’-CAC CAG CCA CTA TTG AAG AAG A-3’ with MANF r1: 5’-AGC ATC ATC GTG GTC CAA-3’; MANF f2: 5’-AAA GAG AAT CGG TTG TGC TAC T-3’ with MANF r2: 5’-CCA GGA TCT TCT TCA GCT CTT T-3’; 18sF 5’-GTA ACC CGT TGA ACC CCA TT-3’ with 18sR 5’-CCA TCC AAT CGG TAG TAG CG-3’.

### Statistical Analysis

The quantitative data were expressed as mean ± SD. Statistical analyses were performed using Student’s *t*-test for two-group comparisons and one-way ANOVA with Bonferroni *post hoc* test for three groups or more. Statistical differences were considered significant at a *p* value of less than 0.05.

## Results

### p58^IPK^ Deletion Does Not Affect Retinal Morphology in Young Adult Mice

**Figure 1:**
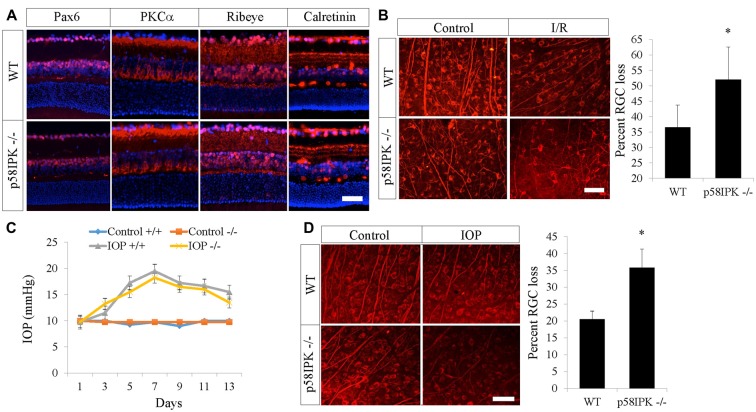
Loss of p58^IPK^ exacerbates retinal ganglion cell (RGC) cell death in retinal ischemia/reperfusion (I/R) and microbead-induced ocular hypertension. **(A)** Retinal cryosections (20 μM) from 2-month-old wild type (WT) and p58^IPK −/−^ mice labeled with primary antibodies against various retinal markers and a fluorescent secondary antibody (red). Anti-Pax6 labels amacrine cells and some RGCs; anti-PKCα labels rod bipolar cells and their dendrites as well as a subset of amacrine cells; anti-Ribeye labels ribbon synapses between bipolar cells and photoreceptors in the OPL, as well as synapses between amacrine cells and RGCs in the IPL; anti-calretinin labels three synaptic lamina within the IPL. There is no clear difference between WT and p58^IPK −/−^ retina in labeling for any of these markers. All sections are counterstained with DAPI (blue) to label nuclei. *n* = 3 mice per group, scale bar = 50 μm. **(B)** Retinal whole mounts from WT and p58^IPK −/−^ mice at 7 days after retinal I/R were stained with RGC marker, Tuj1. Representative images are shown on the left. Graph shows that the percentage of RGC loss, as determined from wholemount retinal Tuj1 staining, is significantly higher in p58^IPK −/−^ mice compared to WT. Mean ± SD, *n* = 5 mice. *p < 0.05 (Student’s *t*-test). Scale bar = 100 μm. **(C)** Graph depicts the average intraocular pressure (IOP) of sham control eyes and microbead-injected eyes in WT (+/+) and p58^IPK −/−^ mice at the time points as indicated. **(D)** Retinal whole mounts from WT and p58^IPK −/−^ mice at 14 days after microbead injection were stained with RGC marker, Tuj1. Representative images are shown on the left. Bar graph shows the percentage of RGC loss is significantly greater in p58^IPK −/−^ mice compared to WT. Mean ± SD, *n* = 4 mice. *p < 0.05 (Student’s *t*-test).

We first examined overall retinal morphology of young adult p58^IPK −/−^ and wild type (WT) mice using a variety of retinal cell type specific markers. We found that there was no significant difference in retinal expression of PKCα, Pax6, ribeye, or calretinin in p58^IPK −/−^ and WT mice (Figure 1A). The retinal layer thickness was identical in these mice (not shown). We conclude that the retinas of p58^IPK −/−^ mice were morphologically indistinguishable from WT at the ages we performed the retinal injury interventions described below.

### p58^IPK^ Deficiency Exacerbates RGC Loss Induced by Retinal I/R

Retinal I/R is a widely used model for investigating the mechanisms and treatment of inner retinal neuron injury caused by acute ischemia. Seven days after I/R, RGC numbers were examined by immunostaining for Tuj1, a putative RGC marker, in retinal whole mounts. We found a 36.6% RGC loss in WT mice and a 52.0% RGC loss in p58^IPK −/−^ mice (Figure 1B). This indicates that I/R induced a significantly higher rate of RGC loss (a 42% increase) in p58^IPK −/−^ mice than in WT, suggesting a protective role of p58^IPK^ in RGCs.

### Loss of p58^IPK^ Increases RGC Damage in Microbead-Induced Ocular Hypertension

We used a second model that is highly relevant to glaucoma to validate the role of p58^IPK^ in RGC protection. We found no significant difference in IOP elevation after microbead injection in p58^IPK −/−^ and WT mice (Figure 1C). Two weeks after induction, WT retinas with increased IOP exhibited a 20% loss of RGCs; the percentage of RGC loss increased significantly to over 35% in p58ipk −/− mice (Figure 1D). This again indicates that a loss of p58^IPK^ results in an increase in RGC death.

### Overexpression of p58^IPK^ Protects RGCs From ER Stress-Induced Cell Death

**Figure 2:**
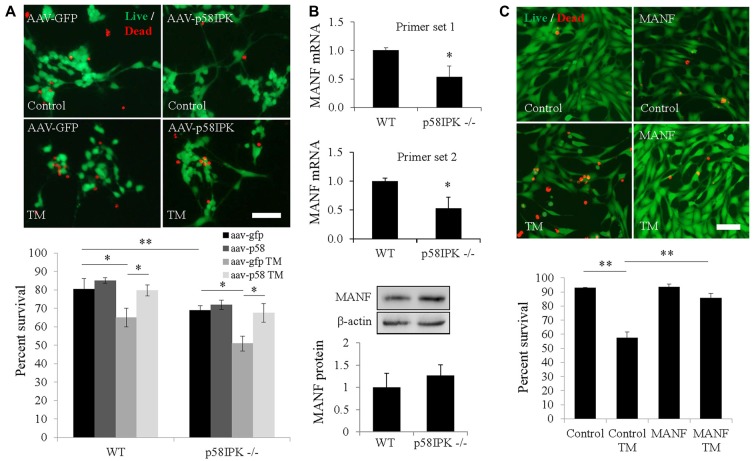
Overexpressing p58^IPK^ by adeno-associated virus (AAV) protects RGCs from endoplasmic reticulum (ER) stress-induced cell death.** (A)** Cultured primary mouse RGCs isolated from p58^IPK −/−^ and WT retinas were infected with AAV-GFP or AAV-p58^IPK^ 24 h prior to a 16 h-treatment with tunicamycin (TM; 1 μg/ml) or control. Cells are labeled with the live/dead cytotoxicity assay (calcein-AM to label live cells (green) and ethidium homodimer-1 to label dead cells (red)). Representative images of live/dead assay are shown above. Graph depicts the survival percentage of RGCs from WT and p58^IPK −/−^ mice. Under control conditions, p58^IPK −/−^ RGCs infected with AAV-GFP have a significantly lower survival percentage than the matched WT group. RGCs from both WT and p58^IPK −/−^ mice infected with AAV-GFP show a significant decrease in survival percentage after TM treatment compared to control treatment. The survival percentage is significantly increased for RGCs infected with AAV-p58^IPK^, compared to infection with AAV-GFP for both WT and p58^IPK −/−^ mice. Mean ± SD, *n* = 3 independent experiments. *p < 0.05 (one-way ANOVA with Bonferroni *post hoc* test). Scale bar = 80 μm. **(B)** Quantitative RT-PCR (qPCR; top) using total RNA from whole retina from 2-month-old WT and p58^IPK −/−^ mice. Two sets of independent primers specific for mesencephalic astrocyte-derived neurotrophic factor (MANF) reveal a mean significant 50% reduction in mRNA level in p58^IPK −/−^ retina compared to WT. All qPCR data are normalized to 18 s. Mean ± SD, *n* = 3 mice per genotype. *p < 0.05 (Student’s *t*-test). Western blot analysis (bottom) of whole retina lysate from WT or p58^IPK −/−^ mice reveal that the level of MANF protein is not statistically different for whole retina (*n* = 6 mice per group). **(C)** R28 cells in culture exposed to 16-h pre-treatment with 50 ng/ml MANF or vehicle, followed by 5 μg/ml TM or control treatment for 24 h. Cells are labeled with the live/dead cytotoxicity assay as in **(A)**. Quantification of the survival of R28 cells reveals that approximately 60% of R28 cells survive TM treatment in the absence of MANF. With MANF pre-treatment the survival percentage is significantly increased to over 85%. Mean ± SD, *n* = 4 independent experiments; *p < 0.05, **p < 0.01 (one-way ANOVA with Bonferroni *post hoc* test). Scale bar = 100 μm.

Next, we examined whether overexpressing p58^IPK^ by AAV is sufficient to protect primary mouse RGCs isolated from p58^IPK −/−^ and WT mice. Under control conditions, we found that the percentage of RGCs survival in culture was significantly decreased in p58^IPK −/−^ RGCs compared to WT (Figure 2A), although this decrease was not rescued by acute overexpression of p58^IPK^. TM exposure caused a significant loss of cells in both p58^IPK −/−^ and WT RGCs infected with control virus (Figure 2A). Overexpressing p58^IPK^ led to a significant recovery to near control levels of survivability in both WT and p58^IPK −/−^ RGCs.

### Reduced Retinal mRNA Expression of MANF in p58^IPK −/−^ Mice

MANF is a member of a newly identified ER-localized neurotrophic factor family and is upregulated during ER stress (Glembotski et al., 2012). We investigated whether MANF expression is altered in p58^IPK −/−^ retinas. Using qPCR, we found that MANF mRNA expression was significantly reduced in the retina of p58^IPK −/−^ mice (Figure 2B). However, the protein level appeared to be insignificantly different (Figure 2B). To further explore whether there is a reciprocal regulation between p58^IPK^ and MANF, we transduced R28 cells with adenovirus to overexpress p58^IPK^ and then treated the cells with TM to induce ER stress. We found that both MANF and p58^IPK^ levels were significantly increased after TM treatment (Supplementary Figure S1A). Overexpression of p58^IPK^ did not alter MANF protein expression in unstimulated cells, but significantly reduced the induction of MANF by TM (Supplementary Figure S1A).

### Recombinant MANF Protects R28 Cells or Retinal Explants From ER Stress Induced Cell Death and Caspase-3 Activation

To examine whether MANF is important for neuronal survival under ER stress, we pretreated R28 cells with 50 ng/ml recombinant hMANF protein or vehicle (0.1% PBS) followed by treatment with 5 μg/ml TM, or vehicle, for 24 h. We find TM treatment induces cell death in 43% of R28 cells in the absence of MANF treatment (Figure 2C). With MANF pretreatment, this number was reduced to 16%, which represents a recovery to 91% of the survival rate of control R28 cells treated with vehicle (Figure 2C). In addition, MANF treatment significantly reduced TM-elicited caspase-3 activation in *ex vivo* cultured retinal explants (Supplementary Figure S1B).

## Discussion

The findings from the present study provide strong evidence for an endogenous role of p58^IPK^ in protection of RGCs in conditions of ER stress, acute ischemia and ocular hypertension. Previous studies, including our own, have implicated p58^IPK^ in a broad range of neurodegenerative diseases and diabetes (Ladiges et al., 2005; Boriushkin et al., 2014, 2015, 2016; Han et al., 2015; McLaughlin and Zhang, 2015). Clinically, patients bearing loss-of-function mutations in the DNAJC3 gene, which encodes p58^IPK^, suffer from diabetes and multisystemic neurodegeneration (Synofzik et al., 2014). In animal models, loss of p58^IPK^ results in increased ER stress and oxidative stress, decreased β cell function, progressive cell death and diabetes in male p58^IPK^ KO mice (Ladiges et al., 2005; Han et al., 2015). In contrast to the relatively thoroughly studied role of p58^IPK^ in diabetes, how p58^IPK^ regulates neuronal function remains unexplored. Herein, we examined the impact of p58^IPK^ deficiency on RGC survival in disease-relevant models. Our data clearly indicate an important role of p58^IPK^ in RGC survival during acute retinal ischemia and in ocular hypertension. Notably, we used young adult p58^IPK^ KO mice that demonstrate no significant morphological defects in the retina compared to their age-matched littermate controls. In addition, most of our experiments were carried out in female mice at 2 months of age, which do not experience increased blood glucose levels as some male p58^IPK^ KO mice do with age.

Our *in vitro* results suggest a therapeutic potential of overexpressing p58^IPK^ or enhancing its function for neuroprotection of RGCs, which warrants an *in vivo* study in disease models. Another interesting finding is that there is likely a reciprocal regulation between p58^IPK^ and MANF in retinal cells undergoing ER stress. Recent studies show that MANF protects CNS neurons (Voutilainen et al., 2017) and markedly improves RGC survival in a rat glaucoma model (Gao et al., 2017). Our data support these findings and demonstrate that MANF inhibits ER stress-induced cell death and caspase-3 activation in retinal cells and explants. In R28 cells, both p58^IPK^ and MANF are upregulated during ER stress; interestingly, overexpressing p58^IPK^ reduces ER stress-stimulated MANF upregulation. This suggests a potential interaction between the two chaperones, which both bind to GRP78 and regulate the UPR, although it remains unclear whether MANF secretion is affected by p58^IPK^ manipulation. Future studies will investigate how MANF and p58^IPK^ are regulated in RGCs and whether overexpression of p58^IPK^ and MANF would synergistically promote RGC survival and function.

## Author Contributions

TM designed and performed the experiments, analyzed the data and drafted the manuscript. ND and JL performed the experiments, analyzed the data and revised the manuscript. JW and SZ conceived and designed the study, reviewed all the data, drafted and revised the manuscript. All authors read and approved the final manuscript.

## Conflict of Interest Statement

The authors declare that the research was conducted in the absence of any commercial or financial relationships that could be construed as a potential conflict of interest.

## References

[B2] Boriushkin E. Wang J. J. Li J. Bhatta M. Zhang S. X. (2016). p58(IPK) suppresses NLRP3 inflammasome activation and IL-1β production via inhibition of PKR in macrophages. Sci. Rep. 6:25013. 10.1038/srep25013 27113095 PMC4845006

[B3] Boriushkin E. Wang J. J. Li J. Jing G. Seigel G. M. Zhang S. X. (2015). Identification of p58IPK as a novel neuroprotective factor for retinal neurons. Invest. Ophthalmol. Vis. Sci. 56, 1374–1386. 10.1167/iovs.14-15196 25655802 PMC4340432

[B1] Boriushkin E. Wang J. J. Zhang S. X. (2014). Role of p58IPK in endoplasmic reticulum stress-associated apoptosis and inflammation. J. Ophthalmic. Vis. Res. 9, 134–143. 24982747 PMC4074489

[B4] Cai M. Wang H. Li J. J. Zhang Y. L. Xin L. Li F.. (2016). The signaling mechanisms of hippocampal endoplasmic reticulum stress affecting neuronal plasticity-related protein levels in high fat diet-induced obese rats and the regulation of aerobic exercise. Brain Behav. Immun. 57, 347–359. 10.1016/j.bbi.2016.05.010 27189035

[B5] Chen C. Cano M. Wang J. J. Li J. Huang C. Yu Q.. (2014). Role of unfolded protein response dysregulation in oxidative injury of retinal pigment epithelial cells. Antioxid. Redox Signal. 20, 2091–2106. 10.1089/ars.2013.5240 24053669 PMC3995121

[B6] Chen H. Wei X. Cho K.-S. Chen G. Sappington R. Calkins D. J.. (2011). Optic neuropathy due to microbead-induced elevated intraocular pressure in the mouse. Invest. Ophthalmol. Vis. Sci. 52, 36–44. 10.1167/iovs.09-5115 20702815 PMC3053285

[B7] Gao F.-J. Wu J.-H. Li T.-T. Du S.-S. Wu Q. (2017). Identification of mesencephalic astrocyte-derived neurotrophic factor as a novel neuroprotective factor for retinal ganglion cells. Front. Mol. Neurosci. 10:76. 10.3389/fnmol.2017.00076 28367115 PMC5355452

[B8] Glembotski C. C. Thuerauf D. J. Huang C. Vekich J. A. Gottlieb R. A. Doroudgar S. (2012). Mesencephalic astrocyte-derived neurotrophic factor protects the heart from ischemic damage and is selectively secreted upon sarco/endoplasmic reticulum calcium depletion. J. Biol. Chem. 287, 25893–25904. 10.1074/jbc.m112.356345 22637475 PMC3406674

[B9] Han J. Song B. Kim J. Kodali V. K. Pottekat A. Wang M.. (2015). Antioxidants complement the requirement for protein chaperone function to maintain β-cell function and glucose homeostasis. Diabetes 64, 2892–2904. 10.2337/db14-1357 25795214 PMC4512214

[B10] Hu Y. Park K. K. Yang L. Wei X. Yang Q. Cho K.-S.. (2012). Differential effects of unfolded protein response pathways on axon injury-induced death of retinal ganglion cells. Neuron 73, 445–452. 10.1016/j.neuron.2011.11.026 22325198 PMC3278720

[B11] Huang C. Wang J. J. Ma J. H. Jin C. Yu Q. Zhang S. X. (2015). Activation of the UPR protects against cigarette smoke-induced RPE apoptosis through up-regulation of Nrf2. J. Biol. Chem. 290, 5367–5380. 10.1074/jbc.m114.603738 25568320 PMC4342454

[B12] Huang X. Wu D. Y. Chen G. Manji H. Chen D. F. (2003). Support of retinal ganglion cell survival and axon regeneration by lithium through a Bcl-2-dependent mechanism. Invest. Ophthalmol. Vis. Sci. 44, 347–354. 10.1167/iovs.02-0198 12506095

[B13] Huber A.-L. Lebeau J. Guillaumot P. Pétrilli V. Malek M. Chilloux J.. (2013). p58IPK-mediated attenuation of the proapoptotic PERK-CHOP pathway allows malignant progression upon low glucose. Mol. Cell 49, 1049–1059. 10.1016/j.molcel.2013.01.009 23395000

[B14] Jiao J. Huang X. Feit-Leithman R. A. Neve R. L. Snider W. Dartt D. A.. (2005). Bcl-2 enhances Ca^2+^ signaling to support the intrinsic regenerative capacity of CNS axons. EMBO J. 24, 1068–1078. 10.1038/sj.emboj.7600589 15719013 PMC554135

[B15] Jing G. Wang J. J. Zhang S. X. (2012). ER stress and apoptosis: a new mechanism for retinal cell death. Exp. Diabetes Res. 2012:589589. 10.1155/2012/589589 22216020 PMC3246718

[B16] Kim S. Joe Y. Jeong S. O. Zheng M. Back S. H. Park S. W.. (2014). Endoplasmic reticulum stress is sufficient for the induction of IL-1β production via activation of the NF-κB and inflammasome pathways. Innate Immun. 20, 799–815. 10.1177/1753425913508593 24217221

[B17] Ladiges W. C. Knoblaugh S. E. Morton J. F. Korth M. J. Sopher B. L. Baskin C. R.. (2005). Pancreatic β-cell failure and diabetes in mice with a deletion mutation of the endoplasmic reticulum molecular chaperone gene P58IPK. Diabetes 54, 1074–1081. 10.2337/diabetes.54.4.1074 15793246

[B18] Lee A. H. Iwakoshi N. N. Glimcher L. H. (2003). XBP-1 regulates a subset of endoplasmic reticulum resident chaperone genes in the unfolded protein response. Mol. Cell. Biol. 23, 7448–7459. 10.1128/mcb.23.21.7448-7459.2003 14559994 PMC207643

[B20] McLaughlin T. Falkowski M. Park J. W. Keegan S. Elliott M. Wang J. J.. (2018). Loss of XBP1 accelerates age-related decline in retinal function and neurodegeneration. Mol. Neurodegener. 13:16. 10.1186/s13024-018-0250-z 29615095 PMC5883257

[B19] McLaughlin T. Zhang S. X. (2015). The neuroprotective potential of endoplasmic reticulum chaperones. Neural Regen. Res. 10, 1211–1213. 10.4103/1673-5374.162696 26487839 PMC4590224

[B21] Rutkowski D. T. Kang S.-W. Goodman A. G. Garrison J. L. Taunton J. Katze M. G.. (2007). The role of p58IPK in protecting the stressed endoplasmic reticulum. Mol. Biol. Cell 18, 3681–3691. 10.1091/mbc.e07-03-0272 17567950 PMC1951758

[B22] Synofzik M. Haack T. B. Kopajtich R. Gorza M. Rapaport D. Greiner M.. (2014). Absence of BiP co-chaperone DNAJC3 causes diabetes mellitus and multisystemic neurodegeneration. Am. J. Hum. Genet. 95, 689–697. 10.1016/j.ajhg.2014.10.013 25466870 PMC4259973

[B23] van Huizen R. Martindale J. L. Gorospe M. Holbrook N. J. (2003). P58IPK, a novel endoplasmic reticulum stress-inducible protein and potential negative regulator of eIF2α signaling. J. Biol. Chem. 278, 15558–15564. 10.1074/jbc.m212074200 12601012

[B24] Voutilainen M. H. De Lorenzo F. Stepanova P. Bäck S. Yu L.-Y. Lindholm P.. (2017). Evidence for an additive neurorestorative effect of simultaneously administered CDNF and GDNF in hemiparkinsonian rats: implications for different mechanism of action. eNeuro 4:ENEURO.0117-16.2017. 10.1523/eneuro.0117-16.2017 28303260 PMC5346176

[B25] Zhang S. X. Sanders E. Fliesler S. J. Wang J. J. (2014). Endoplasmic reticulum stress and the unfolded protein responses in retinal degeneration. Exp. Eye Res. 125, 30–40. 10.1016/j.exer.2014.04.015 24792589 PMC4122592

[B26] Zhong Y. Li J. Chen Y. Wang J. J. Ratan R. Zhang S. X. (2012). Activation of endoplasmic reticulum stress by hyperglycemia is essential for muller cell-derived inflammatory cytokine production in diabetes. Diabetes 61, 492–504. 10.2337/db11-0315 22228718 PMC3266398

