# Supplementary material for: p58IPK Is an Endogenous Neuroprotectant for Retinal Ganglion Cells
Source: Front Aging Neurosci. 2018 Sep 7;10:267. doi: 10.3389/fnagi.2018.00267 (PMC6137320; doi:10.3389/fnagi.2018.00267)
Supplement: Supplementary file 1 [file Data_Sheet_1.PDF]

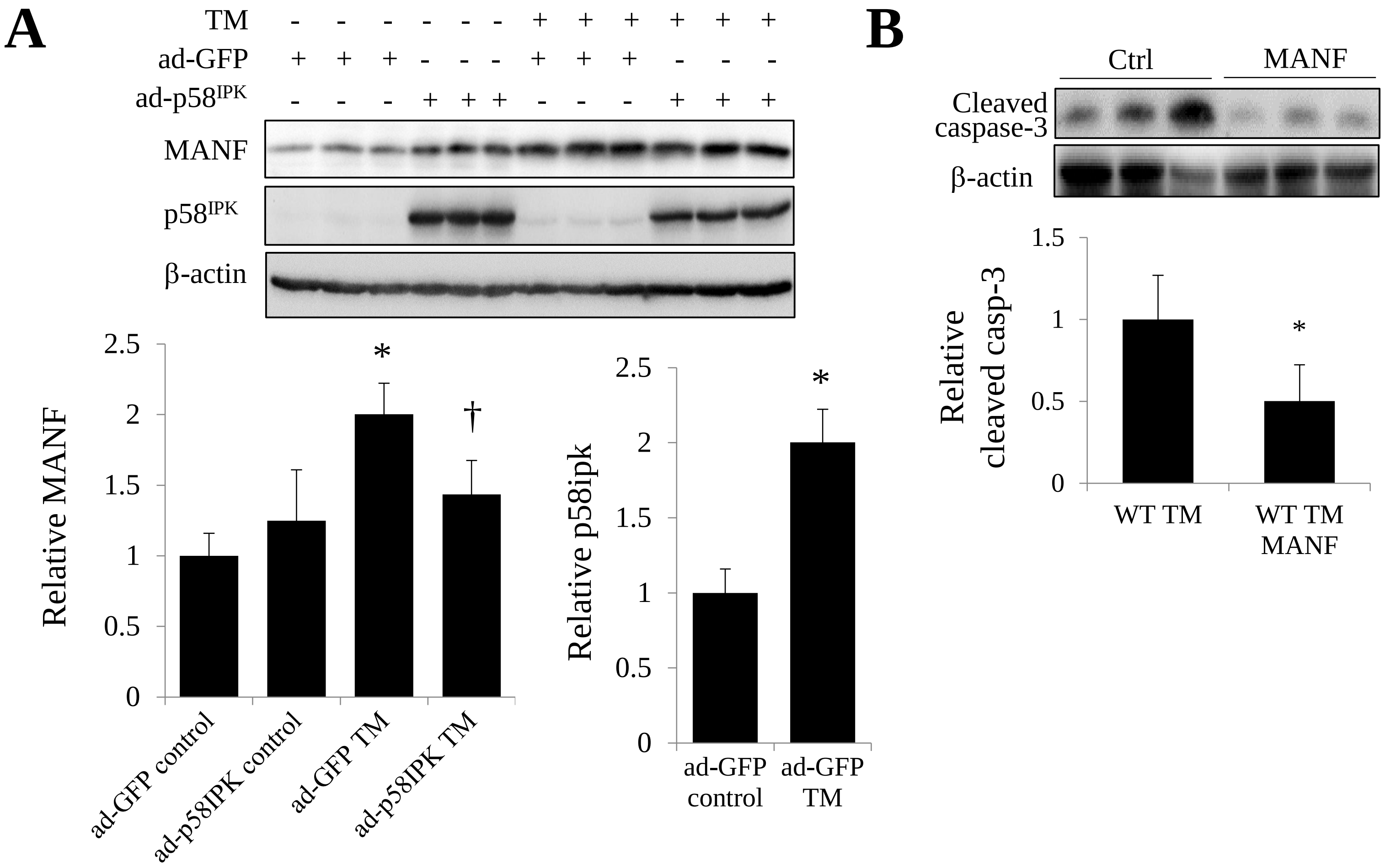

Suppl. Fig. 1. A) ER stress induces increased protein expressions of MANF and p58ipk in R28 cells. Cells were transduced with adenovirus expressing GFP (ad-GFP) as control or p58^{ipk} (ad-p58^{ipk}) 24 hours prior to treatment with 1µg/ml TM or vehicle for an additional 24 hours. Western blots reveal that both MANF and p58ipk levels are significantly increased with TM treatment in ad-GFP transduced cells. There is no significant difference in MANF level between ad-GFP controls and ad-p58^{ipk} controls. TM induced a significant 2-fold increase in MANF level in ad-GFP transduced cells, but to a lesser extent in ad-p58ipk transduced cells. Results represent 4 independent experiments. * $p < 0.05$ vs. ad-GFP control; † $p < 0.05$ vs. ad-GFP TM; one-way ANOVA with Bonferroni post-hoc test. **B)** MANF reduces ER stress-induced caspase 3 activation in mouse retinal explants. Mouse retinal explants were incubated with 5µg/ml tunicamycin (TM) as control (Ctrl) or 5µg/ml TM plus 50ng/ml MANF for 14 hours. Retina lysate were subjected to Western blot analysis. Results show that MANF treatment significantly reduces the relative level of the apoptotic marker, cleaved-caspase-3. n=3 mice per group. * $p < 0.05$, Student's *t* test.
