# Supplementary material for: p58IPK Is an Endogenous Neuroprotectant for Retinal Ganglion Cells
Source: Front Aging Neurosci. 2018 Sep 7;10:267. doi: 10.3389/fnagi.2018.00267 (PMC6137320; doi:10.3389/fnagi.2018.00267)
Supplement: Supplementary file 2 [file Data_Sheet_2.PDF]

Supplemental Methods

Mouse RGC isolation and culture

Retinal ganglion cells (RGCs) were isolated using the Miltenyi Biotec magnetic cell sorting (MACS) system as described previously (Huang et al., 2003;Jiao et al., 2005). Briefly, retinas from 4 or 5 neonatal (postnatal day 6) mice of the same genotype were dissected immediately after sacrifice and cut into small pieces with micro-scissors. Retinal pieces were digested at 37 ˚C with 0.5 mg/ml dispase, 0.5 mg/ml collagenase, and 100 µg/ml DNase in HBSS for 15-30 minutes with periodic agitation. An equal volume of media was added and retina dissociated by gentle pipetting prior to low-speed centrifugation at 4 ˚C. Dissociated cells were re-suspended in PBS with 2 mM EDTA, and 0.5% BSA (MACS buffer) and incubated with 30 µl magnetic bead-conjugated Thy1.2 antibody (CD90.2, Miltenyi Biotec) at 4 ˚C for 15 min. The cells were sorted by passage through a magnetic column attached to a magnet per manufacturer's instructions (Miltenyi Biotec). Cells bound by magnetic bead-conjugated antibody remain within the column and are recovered and re-suspended by flushing the column with MACS buffer after removing the column from the magnet. After two rounds of low-speed centrifugation and washes in MACS buffer, the RGC pellet was re-suspended in media (Neurobasal-A medium supplemented with 20 µl/ml 50X B-27 Supplement, 100 U/ml penicillin–streptomycin, 2 mM glutamine, 25 µM glutamate, 50 ng/ml brain-derived neurotrophic factor, 50 ng/ml ciliary neurotrophic factor, 5 ng/ml forskolin, and 5 µg/ml insulin). Cell solution was immediately incubated with either AAV-GFP (complete viral particles purchased from Virovek, Inc.) or AAV-p58^{ipk} (complete viral particles

purchased from Vector Biolabs) at 10^12 GC/ml for 15 minutes. Transduction rates in preliminary experiments were found to be between 60 - 80%, based on GFP labeling, for 10^12 GC/ml. RGCs were seeded in tissue culture plates coated with poly-D-lysine (10 µg/ml) and laminin (10 µg/ml) and cultured at 37 ˚C and 5% CO_2 for 24 h. The cells were then treated with 1 µg/ml tunicamycin (TM), or vehicle, for an additional 16 h prior to cell viability analysis.

RGC enrichment was initially determined by fixing cultures with 4% paraformaldehyde for 15 min at 37 ˚C, followed by PBS wash. Fixed cells were incubated with PBS, 1% Triton X-100, and 1% BSA for one hour, followed by incubation with the TuJ1 antibody (anti-neuronal class III β-tubulin, rabbit, Covance MRB-435P) at 1:1000 overnight at 4 ˚C. After washing 3 times with PBS plus 1% Triton X-100, Texas-red conjugated goat-anti-rabbit secondary antibody (1:800; ThermoFisher T-6391) was applied for 1 hour, rinsed, counterstained with DAPI to label nuclei, and examined under fluorescence microscope.

Cell viability analysis

Cell viability was assessed by the live/dead cytotoxicity assay (Molecular Probes) following the manufacturer's instruction. Briefly, cells were incubated with a solution comprised of 4 µM calcein AM and 4 µM ethidium homodimer-1 in PBS for 15 min at 37 ˚C. Cells were photographed while remaining in the live/dead labeling solution under fluorescent illumination on a Zeiss AxioVert microscope using a 20x objective. Between 5-8 images were taken per condition and genotype for each experiment, choosing fields with minimal cell clumping. Images were assessed for live cells (green) and dead cells

(red), blind to treatment or genotype. Red fluorescence is due to ethidium homodimer-1, which is excluded from cells with an intact plasma membrane, entering cells with damaged membranes and fluorescing upon binding nucleic acids. The green fluorescence is from cell-permeant calcein-AM, which is non-fluorescent until converted to intensely fluorescent calcein (which is retained in live cells) by ubiquitous intracellular esterase activity. The calcein fluorescence is significantly more intense than GFP in these cells and, in addition, GFP is never present in cells which are labeled with ethidium homodimer-1 (i.e. dead cells). Thus, every cell labeled green is counted as alive and every cell labeled red is counted as dead. For each of the four conditions analyzed for each genotype the average survival rate was calculated for each trial as the average of all living cells divided by the living cells plus dead cells for each group.

Mouse retinal ischemia/reperfusion model

Mice were anesthetized with 150 mg/kg ketamine and 7.5 mg/kg xylazine. Pupils were dilated with 1% atropine sulfate (Falcon) and 2.5% phenylephrine hydrochloride (Akorn). The anterior chamber was canulated with 30g needle attached by sterile tubing to a 60ml reservoir of sterile PBS suspended approximately 5 feet above the mouse. Ocular pressure was recorded by bounce tonometer (Icare) every several minutes until stable and then periodically until the end of the application. A consistent pressure in excess of 90 mmHG was maintained for 50-60 minutes. Retinas were re-perfused by careful removal of the needle from the anterior chamber. After 7 days, mice were sacrificed and the eyes removed and processed for retinal wholemount TuJ1 staining as described below. Each mouse had one eye at high pressure and one eye at control pressure (10 mmHg). The “n =

5” represents 5 mice, each with one control eye and one experimental eye.

Mouse microbead-induced ocular hypertension model

Mice were anesthetized with 100 mg/kg ketamine and 5 mg/kg xylazine. Pupils were dilated with ophthalmic drops containing 1% atropine sulfate (Falcon) and 2.5% phenylephrine hydrochloride (Akorn). The anterior chamber was canulated with a 33g needle attached to a syringe loaded with 2 µl PBS containing 10 µm diameter polystyrene, fluorescent microspheres at 1.5 x 10^7 beads/ml (Invitrogen, FluoSpheres F8830). Bead solution was injected into the anterior chamber and the mouse positioned to recover on its side with the bead-injected eye facing up. Ocular pressure was recorded by bounce tonometer (Icare) prior to injection and every 2 days thereafter at the same time of day. Mice with pressure between 15-23 mmHG for at least 5 days were processed. Two weeks after microbead injection mice were sacrificed and processed for retinal wholemount TuJ1 staining as described below. The “n = 4” represents 4 mice, each with one control eye and one experimental eye.

TuJ1 labeling of retinal wholemounts

Eyes were removed immediately after sacrifice and immersion fixed in 4% paraformaldehyde for 1 hour and then washed with PBS several times. Retinas were then dissected out as whole retinal eye cups. Retinal cups were incubated with blocking solution (PBS, 1% Triton X-100, and 1% BSA) for one hour, followed by incubation with an anti-TuJ1 antibody (anti-neuronal class III β-tubulin, rabbit, Covance MRB-435P) at 1:1000 in blocking solution overnight at 4 ˚C with mild agitation. After

incubation, retinas were washed 3 times with PBS plus 1% Triton X-100 and Texas-red conjugated goat-anti-rabbit secondary antibody (1:800 in blocking solution; ThermoFisher T-6391) was applied overnight at 4 °C and again washed three times with PBS plus 1% Triton X-100. Retinas were mounted flat by making radial relief cuts at 3-5 positions around the retinal cup from the periphery to near the optic disc and coverslipped for fluorescent photography. Five photographs with the focal plane centered on the GCL were taken for each retina using a 20X objective on an upright Olympus BX53 microscope and DP80 camera. Images were taken between 50-70% of the radial distance from the optic disc to the peripheral edge of the retina from at least 3 quadrants and in locations devoid of major blood vessels. All TuJ1-positive cell bodies within the ganglion cell layer in each image were counted by an independent observer blind to condition and genotype. From each animal one control retina and one experimental retina were processed. All five images for each control retina and each experimental were averaged and percent loss calculated for each animal by dividing the average number of RGCs per field in the experimental eye by the average number of RGCs per field in the control eye. The n values represent the number of mice used for each condition.

***Ex vivo* retinal explant culture and treatment**

Mouse retinas were dissected in HBSS immediately after sacrifice and incubated in Neurobasal-A media supplemented with 20 µl/ml 50X B-27 Supplement, 2 mM glutamine, 25 µM glutamate, 50 ng/ml brain-derived neurotrophic factor, 50 ng/ml ciliary neurotrophic factor, 5 ng/ml forskolin, and 5 µg/ml insulin. After 6 hours at 37 °C and 5% CO_2, one retina from each mouse was treated with 5 µg/ml TM and the other retina treated with 5µg/ml TM plus 50 ng/ml MANF and cultured for an additional 14 hours.

Retinas were washed two times in ice cold PBS. Total protein was isolated and subjected to Western blot analysis as described below.

Adenoviral transduction of R28 cells

R28 cells were treated with adenovirus expressing murine $p58^{IPK}$ (ad-$p58^{IPK}$) or GFP (ad-GFP). Generation and amplification of the adenoviruses were described previously (Boriushkin et al., 2015). R28 cells were transduced with the viruses at an MOI of 50 and incubated for 24 hours and then treated with 1 µg/ml TM for an additional 24 hours. Protein was isolated from R28 cells as described below for Western blot analysis.

Western blot analysis

Retinas were dissected and flash frozen in liquid nitrogen. Frozen retinal tissue was thawed on ice in chilled fresh radio immune precipitation assay (RIPA) buffer with protease inhibitor mixture, PMSF, and sodium orthovanadate and sonicated. R28 cells were chilled on ice, washed with PBS, and lysed in RIPA plus protease inhibitors. Each well of R28 cells on a 6-well plate was collected using a cell scraper prior to sonication. Protein samples (approximately 25-35 µg), MagicMark XP Western Protein Standard (Life Technologies, LC5602), and BenchMark Prestained Protein Ladder (Life Technologies, 10748-010) were electrophoresed in 12% SDS-PAGE resolving gels and transferred to nitrocellulose blotting membranes (Pall Corporation) at a constant 0.34 Amps for 3.25 hours. Membranes were incubated with the appropriate primary antibody (anti-MANF, Sigma SAB3500384; anti-$p58^{IPK}$, Cell Signaling C56E7; anti-cleaved-caspase-3, Cell Signaling 9664) at 1:1000 in TBST [Tris-buffered saline plus 0.1%

TWEEN 20 (Sigma)] plus 3% BSA overnight at 4 ˚C. After multiple washes with TBST membranes were incubated with peroxidase conjugated secondary antibody (Vector PI-1000, anti-rabbit) at 1:10000 in TBST plus 3% BSA for 1 hour at room temperature before development with SuperSignal West Dura Extended Duration Substrate (Thermo Scientific, #34076) per manufacturer's instructions. Blots were imaged with a Bio-Rad ChemiDoc MP imaging system with ImageLab software. Membranes were re-blotted with an anti-β-actin antibody (Abcam ab8226, 1:10000 in TBST plus 3% BSA, overnight at 4 ˚C) and then with a peroxidase conjugated anti-mouse secondary antibody (Vector PI-2000) as loading control. Bands were normalized by densitometry using ImageLab software to the β-actin band.

Quantitative RT-PCR (qPCR)

Total RNA was extracted from tissue homogenized through multiple passes through a 27G needle with Trizol (Invitrogen) following the manufacturer's instructions. Quantity and quality of total RNA was determined by spectrophotometry using NanoDrop 2000 (ThermoFisher). The iScript cDNA Synthesis Kit (Bio-Rad) was used for cDNA synthesis per manufacturer's instructions. Quantitative RT-PCR was performed using the iQ SYBR Green Supermix (Bio-Rad) per manufacturer's instructions. Normalization was performed using 18S ribosomal RNA as an endogenous control. The PCR products of all primer pairs were verified as single bands of the predicted size prior to use.

References

Boriushkin, E., Wang, J.J., Li, J., Jing, G., Seigel, G.M., and Zhang, S.X. (2015). Identification of p58IPK as a Novel Neuroprotective Factor for Retinal Neurons. *Invest Ophthalmol Vis Sci*, doi:10.1167/iovs.1114-15196

Huang, X., Wu, D.Y., Chen, G., Manji, H., and Chen, D.F. (2003). Support of retinal ganglion cell survival and axon regeneration by lithium through a Bcl-2-dependent mechanism. *Invest Ophthalmol Vis Sci* 44**,** 347-354.

Jiao, J., Huang, X., Feit-Leithman, R.A., Neve, R.L., Snider, W., Dartt, D.A., and Chen, D.F. (2005). Bcl-2 enhances Ca(2+) signaling to support the intrinsic regenerative capacity of CNS axons. *EMBO J* 24**,** 1068-1078.
